# Carbon Dioxide, Odorants, Heat and Visible Cues Affect Wild Mosquito Landing in Open Spaces

**DOI:** 10.3389/fnbeh.2018.00086

**Published:** 2018-05-07

**Authors:** Yang-Hong Zhou, Zhong-Wei Zhang, Yu-Fan Fu, Gong-Chang Zhang, Shu Yuan

**Affiliations:** College of Resources, Sichuan Agricultural University, Chengdu, China

**Keywords:** mosquito landing, CO_2_ mimics, heat, humidity, black color

## Abstract

CO_2_ and other chemicals affect mosquito blood meal seeking behavior. Heat, humidity and black color can also serve as orientation cues. However mosquito attraction does not necessarily mean that it will land. The sequence of the cues used for mosquito landing is unclear. We performed a field study with wild mosquitoes in an open space and found that no chemicals (except pyrethrins) could completely prevent mosquitoes from landing. CO_2_ mimics cyclopentanone and pyridine attracted mosquitoes but did not lead to landing. No mosquito was caught in the absence of heat, although in the presence of CO_2_. Mosquito females commonly explore visible black objects by eyes, which is independent of infrared radiation. Humidification around the heat source may increase the detection distance but it did not affect mosquito landing. If a black object was located distant from the CO_2_ and heat, mosquitoes still explored the heat source. Relative to CO_2_ and heat, odorants, humidity and black color show lesser effects on mosquito landing.

## Introduction

Mosquitoes transmit pathogens such as the malaria parasite, Dengue virus and the Zika virus (Yuan et al., 2017). Female mosquitoes use multiple cues to identify and move toward the hosts. These include exhaled CO_2_ (Lacey and Cardé, 2011; Turner et al., 2011; Tauxe et al., 2013; Lacey et al., 2014; McMeniman et al., 2014; van Breugel et al., 2015), skin odors (Eiras and Jepson, 1994; Hallem and Carlson, 2006; Saito et al., 2009; Syed and Leal, 2009; Carey et al., 2010; Turner et al., 2011; Tauxe et al., 2013; McMeniman et al., 2014; Gonzalez et al., 2015), heat (Burgess, 1959; Davis and Sokolove, 1975; Gingl et al., 2005; van Breugel et al., 2015; Zermoglio et al., 2017), humidity (Burgess, 1959; Eiras and Jepson, 1994; van Breugel et al., 2015) and colors (Bidlingmayer and Hem, 1980; Browne and Bennett, 1981; Muir et al., 1992; Gibson and Torr, 1999; Bentley et al., 2009; van Breugel et al., 2015).

The compound 1-octen-3-ol elicits a positive effect to CO_2_-sensing neurons (Gonzalez et al., 2015), while 1-butanal, 1-hexanol, ethyl pyruvate and methyl pyruvate inhibit olfactory receptor activities (Turner et al., 2011; Tauxe et al., 2013). Among all active odor chemicals, 2,3-butanedione causes an unusual ultra-prolonged activation of CO_2_-detecting neurons and thus disrupts CO_2_-mediated activation as well as source-finding behaviors in mosquitoes, even after the odor is no longer present (Turner et al., 2011). However, 2,3-butanedione is a relatively toxic compound (mammalian median lethal dose LD_50_ = 1580 mg/kg), which limits its practical usefulness. The compound 2,3-pentanedione (LD_50_ > 2.5 g/kg) has a similar chemical property but is much less toxic than 2,3-butanedione (Sawyer et al., 1991). The roles of these odor chemicals on mosquito landing were investigated in this study.

A previous study suggested that cyclopentanone mimics the electroantennogram responses induced by CO_2_, and therefore can lure mosquitoes to traps in the absence of carbon dioxide (Tauxe et al., 2013). Ethyl pyruvate and methyl pyruvate, in contrast, strongly inhibit the activity of olfactory receptor neurons (Tauxe et al., 2013). The roles of CO_2_ mimics (in the absence of carbon dioxide) on mosquito landing were tested in this study.

Most previous experiments used lab-reared mosquitoes that were tested in one-way or two-way enclosed tunnels (Lacey and Cardé, 2011; Lacey et al., 2014; van Breugel et al., 2015). The behavior of wild mosquitoes in natural open spaces surrounded with complex lures is less well known. Attraction of a mosquito does not necessarily mean that it will land on a trap or other surfaces. We lack understanding of the importance ranking of cues involved in mosquito landing. Here we studied wild mosquitoes and used a sticky pad/trap catches to infer landing preferences, but not behavioral preferences or near-source behaviors. Besides, both lab-reared and wild mosquitoes are prone to exploring dark objects and moist heat sources (Burgess, 1959; Bidlingmayer and Hem, 1980; Browne and Bennett, 1981; Muir et al., 1992; Gibson and Torr, 1999; McMeniman et al., 2014; van Breugel et al., 2015). The influences of heat, humidity and black color on mosquito landing were also investigated in this field study.

## Materials and Methods

### Animals

The behaviors of wild mosquitoes (mostly *Anopheles sinensis*, *Anopheles lesteri*, *Culex fatigans*, *Culex tritaeniorhymchus* and *Aedes albopictus*) were studied. *Aedes albopictus* only appeared in late summer and early autumn, and therefore was not counted. We counted insect bodies, no matter if their legs were missing. The experiments were performed from June 1st to September 1st in 2015, 2016 and 2017, when the mosquitoes were most active. Usually 9–17 *Anopheles* and 30–56 *Culex* were caught on each sticky plate per night and the numbers of *Anopheles* and *Culex* did not significantly change from June 1st to September 1st each year (Supplementary Figure S1).

### Field Trapping

**Figure 1:**
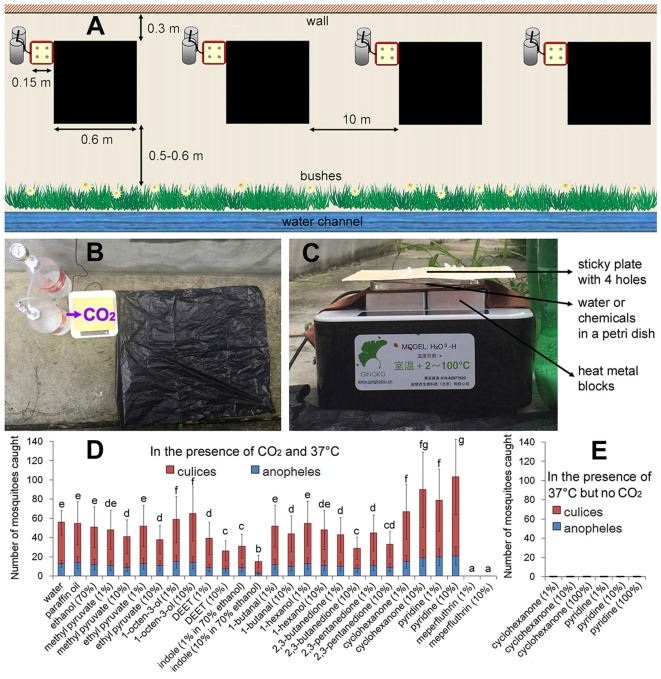
The field trapping device with different odorant chemicals. **(A)** Four independent traps (including one basic trap) about 10 m apart were set simultaneously on 1 day. The basic trap: four metal blocks were heated to 37.0°C, a petri dish of water was placed directly above the blocks, and then a faint-yellow 15 cm × 15 cm sticky plate with four 1 cm diameter holes was placed above. A simple carbon-dioxide generator with sodium citrate and sodium bicarbonate was placed on the left side of the sticky plate. A black polyethylene bag (60 cm × 60 cm) was placed adjacent to the right side of the sticky plate. The mosquito traps were placed in a ventilated corridor near the building (0.3 m to the wall of the building; 0.5–0.6 m to the bushes adjacent next to a drainage channel). **(B)** Top view of the basic trap. **(C)** Lateral view of the basic trap. **(D)** Effects of odorant chemicals on mosquito capture rate. Water refers to the basic trap presenting in addition a Petri dish with water. **(E)** Cyclopentanone and pyridine treatments in the absence of CO_2_ resulted in a zero capture rate. Error bars show standard deviations (*n* = 20 for the basic trap; *n* = 5 for the others). Significant differences are indicated by different lowercase letters.

The field experiment was performed at the Wenjiang Campus of Sichuan Agricultural University in Chengdu (30°41’N, 103°49’E at an altitude of 558 m). The mosquito traps were placed in a ventilated corridor near the third teaching building (0.3 m to the wall of the building; 0.5–0.6 m to the bushes adjacent next to a drainage channel; Figure 1) from 8:00 p.m. to 8:00 a.m. of the next day. The light intensity during the trapping times ranged from about 10–50 μmol photons m^−2^ s^−1^ and the temperature ranged from 18°C to 25°C. A transparent umbrella prevented the trap from getting wet during rainfall.

### The Basic Trap

Four metal blocks were heated to 37.0°C, a petri dish of water (without a lid) was placed directly above the blocks, and then a faint-yellow 15 cm × 15 cm sticky plate (designed for house flies) with four 1 cm diameter holes was placed above. A simple carbon-dioxide generator with sodium citrate and sodium bicarbonate was placed on the left side of the sticky plate (outlet of the hose faced the sticky plate) with a gas flow of about 500 mmol h^−1^. A black polyethylene bag (60 cm × 60 cm) was placed adjacent to the right side of the sticky plate (Figure 1).

### Chemical Treatments

Attractive odor compound 1-octen-3-ol, persistent CO_2_-activation odor chemicals 2,3-butanedione and 2,3-pentanedione, repellents 1-butanal, 1-hexanol, ethyl pyruvate and methyl pyruvate, contact-repellents indole and DEET (N, N-diethyl-meta-toluamide), CO_2_ mimics cyclopentanone and pyridine and an insecticide meperfluthrin were tested. All chemicals were dissolved at 10^−1^ or 10^−2^ in paraffin oil or water (Tauxe et al., 2013), except for indole which was dissolved in 70% ethanol (Gonzalez et al., 2015). The 50 mL chemical solutions were added to the petri dish of each trap. All chemicals were purchased from Sigma-Aldrich Company (St. Louis, MO, USA).

### Variations to the Basic Trap

The metal blocks were heated to 25.0, 37.0 or 50.0°C, respectively. To achieve a dry heat plume, a petri dish without water was placed on the metal blocks with a sticky plate, without holes, above them. We used a humidifier to increase the humidity above the trap. In some experiments, the faint-yellow sticky plate (max reflectance across 250–750 nm ≈65%) was replaced by a black sticky plate (max reflectance of 32%) and the black polyethylene bag (max reflectance of 33%) was replaced by a white paper (60 cm × 60 cm; max reflectance of 67%) with or without a black square (15 cm × 15 cm) at the center, a black polyethylene bag with a white square (15 cm × 15 cm) at the center, a faint-yellow sticky plate, or a black sticky plate. These treatments are indicated in the figures. For the nearly darkness treatment, a black umbrella was placed above the trap to restrict illumination to less than 1 μmol photons m^−2^ s^−1^. Five independent replicates (on different nights) were performed for each varied trap.

### Temperature Measurements IR Thermal Imaging and Sunlight Reflectance Measurements

Infrared radiation photos of the traps were taken by a FLIR T620 thermal-imaging camera (Thermal CAM-FLIR Systems, USA; Zhang et al., 2016). The ambient temperature was set at 21.1°C. Temperatures of the thermal plume created by the heated trap at distances of 0–20 cm (away from the sticky plate) were measured with a digital thermometer (van Breugel et al., 2015). The sunlight reflectance across 250–750 nm range for the different objects was measured by using a reflectance meter (RCRM01, Rinch Industrial Company, China).

### Statistical Analysis

All experiments were performed randomly across months and years. Significant differences among different traps or different treatments were analyzed according to Duncan’s multiple range test at the 5% level. ANOVA was performed by using the software package SPSS v22.0. The experiments were also approved by the Ethics Committee of Sichuan Agricultural University.

## Results

### The Stability Test

The number of mosquitoes present in an outdoor area can vary greatly and it is affected by mosquito species, locations, habitats, weather and other environmental factors. Therefore, we performed a stability test with the basic trap. Based on the standard deviation of 20 independent replicates in 20 different calm and rainless days (from June 1st to July 10st in 2015), we determined that the basic trap should catch 9–17 *Anopheles* and 30–56 *Culex* per night (8:00 p.m. to 8:00 a.m.) under our specific field trapping conditions. Thus for the subsequent experiments, four independent traps about 10 m apart, including one basic trap, were set at the same time on each day (Figure 1A). If the basic trap caught fewer or more mosquitoes than the above thresholds, the data of that day was rejected because of heavy rain, strong wind, or human disturbances. From June 1st to September 1st in 2015, 2016 and 2017 (279 days), 207 of 214 calm and rainless days met this criterion and their month-by-month and year-by-year differences were not significant (Supplementary Figure S1). Therefore seasonal or yearly effects could be ruled out.

### Effects of Odorant Chemicals on Mosquito Landing

Attractive odor compounds or repellents resulted in higher or lower numbers of mosquitoes caught by the traps, respectively (Figure 1D). 2,3-butanedione or 2,3-pentanedione causes an unusual ultra-prolonged activation of CO_2_-detecting neurons and thus disrupts CO_2_-mediated source-finding behaviors in mosquitoes (Turner et al., 2011). Although 50% declines were observed for these two chemicals (Figure 1D), neither 2,3-butanedione nor 2,3-pentanedione can prevent mosquitoes from finding the CO_2_ source. Indole and DEET (N,N-diethyl-meta-toluamide) are mosquito contact-repellents (Gonzalez et al., 2015). However, a large number of mosquitoes were still caught on the sticky plates in the presence of either indole or DEET (Figure 1D).

A previous study indicated that cyclopentanone and pyridine mimic the electroantennogram effects of CO_2_, and therefore are able to attract mosquitoes in large numbers in the absence of carbon dioxide (Tauxe et al., 2013). However in our experimental system, no mosquitoes were caught by using these two chemicals at 37°C but without CO_2_ (Figure 1E). Although they attracted mosquitoes (many mosquitoes hovered 5–20 cm over the traps), neither cyclopentanone nor pyridine could replace the role of CO_2_ in mosquito landing. Among all chemicals tested, only meperfluthrin (a volatile pyrethrin insecticide) treatments resulted in a zero capture rate (Figure 1D).

### Effects of the Black Color on Mosquito Landing

**Figure 2:**
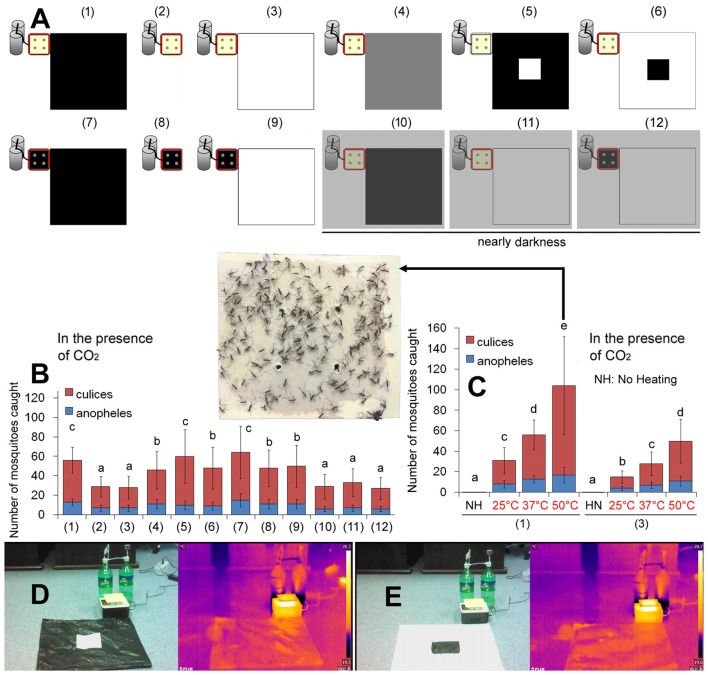
Mosquitoes are attracted to heated black objects. **(A)** Variations to the basic trap (see “Materials and Methods” section for details). **(B)** Capture rates of 12 different traps as indicated in **(A)**. **(C)** Higher temperatures caught more mosquitoes, and no heating resulted in a zero capture rate. The Trap (1) and Trap (3) were used. A representative sticky plate of the basic trap [Trap (1)] at 50°C is shown. Error bars show standard deviations (*n* = 20 for the basic trap; *n* = 5 for the others). Significant differences are indicated by different lowercase letters.** (D)** The Trap (5) presenting a black polyethylene bag with a white square at the center. Its infrared thermal image is displayed on the right pane.** (E)** The Trap (6) presenting a white paper with a black square at the center. Its infrared thermal image is displayed on the right pane.

A previous report found that when mosquitoes were exposed to a CO_2_ plume, they spent much of their time exploring a dark visual feature on a gray background (van Breugel et al., 2015). However it is unclear whether they were attracted to the dark color or to a black and white high-contrast graphic pattern. Figures 2A,B indicates that a large area of black color got the highest capture rate; a large area of white color got the lowest capture rate; while the same area of 50% gray color got a medium rate. A black square on a large area of white caught significantly fewer mosquitoes than a white square on a large area of black did. Thus the larger the area of black color near the CO_2_ source is, the more mosquitoes are trapped. Mosquitoes seem to be more attracted to dark objects than to black and white patterns.

We studied how mosquitoes find dark objects. In nearly darkness (illumination <1 μmol photons m^−2^ s^−1^), white objects and black objects had similar capture rates (Figures 2A,B), which implies that a visual cue is involved. Mosquitoes cannot use infrared radiation (IR) as an orientation cue (Gingl et al., 2005; Zermoglio et al., 2017). We found that white objects and black objects present similar IR thermal images (Figures 2D,E).

### Effects of Heat on Mosquito Landing

When the mosquitoes encounter a CO_2_ stimulus, it can lead to higher levels of attraction to visual and sensory objects (McMeniman et al., 2014; van Breugel et al., 2015). In the current study, we used the sticky plate as the visual cue when testing the impacts of heat. Although the visual target is faint-yellow, it might be a high-contrast visual object to the mosquito. To rule out possible visual disturbance, we tested the basic trap but removed the black polyethylene bag. Interestingly, the faint-yellow sticky plate alone [Trap (2) in Figure 2A] got a capture rate similar to the faint-yellow sticky plate adjacent to a white paper [Trap (3) in Figure 2A]. Considering that the floor of the ventilated corridor also is faint-yellow, very close to the color of the sticky plate (reflectance difference <2%; Supplementary Figure S2), the solo faint-yellow sticky plate may not be a high-contrast visual object to the mosquito. In other words, the Trap (2) in Figure 2A may present a heat cue alone without a visual object.

With these traps of different colors, we found that although black color caught more mosquitoes than the white color, the temperature of the heat source had a greater influence on mosquito landing. No mosquito was caught in the absence of heat, although some mosquitoes hovered 3–20 cm over the CO_2_ outlet (Figure 2C). Temperature of the heat source appears to play a much more important role in mosquito landing than the black color.

### Effects of Humidity on Mosquito Landing

**Figure 3:**
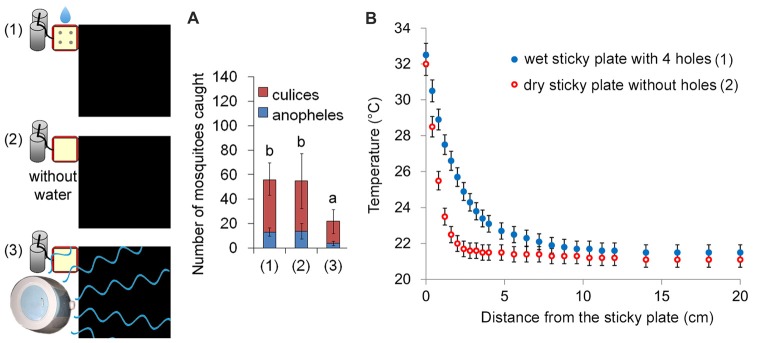
Humidity may affect the detection distance, but not mosquito landing. **(A)** Capture rates of three different traps of a wet sticky plate with four holes [Trap (1); the basic trap], a dry sticky plate without holes and water [Trap (2)] and a humidifier alongside a dry sticky plate respectively. The humidifier was associated with a lower capture rate, because the water vapors may disturb mosquito’s orientation behavior. Error bars show standard deviations (*n* = 20 for the basic trap; *n* = 5 for the others). Significant differences are indicated by different lowercase letters.** (B)** Thermal signatures of the wet heated sticky plate and the dry heated sticky plate. Temperatures away from the sticky plate (above the heated metal blocks) were measured at an ambient temperature of 21.1°C. The metal blocks were heated to 37.0°C.

Besides CO_2_, heat and the black color, mosquitoes had a significantly stronger response to moist and heated objects (van Breugel et al., 2015). However, for the capture rate (mosquito landing), the wet heat source (relative humidity at 10 cm above the sticky plate ≈80%) and the dry heat source (environment relative humidity ≈30%) caught almost the same numbers of mosquitoes. A humidifier alongside a dry sticky plate increased the relative humidity to about 80% (at 10 cm above the sticky plate), but resulted in a lower capture rate (Figure 3), because the water vapors may disturb mosquito orientation behaviors. To determine the spatial scale over which wet/dry thermal cues could realistically be detected by a mosquito, we measured the temperatures away from the sticky plate above the heated metal blocks at an ambient temperature of 21.1°C. At a distance of 10–15 cm, the difference between the dry heat source and ambient temperature was less than 0.2°C, which is the detection threshold for *Aedes* (Davis and Sokolove, 1975). However the distance was greater than 20 cm to the wet heat source (Figure 3). Therefore, humidity affected the detection distance, but did not have an influence on mosquito landing.

### Behavioral Responses of Mosquitoes to Separated Cues

**Figure 4:**
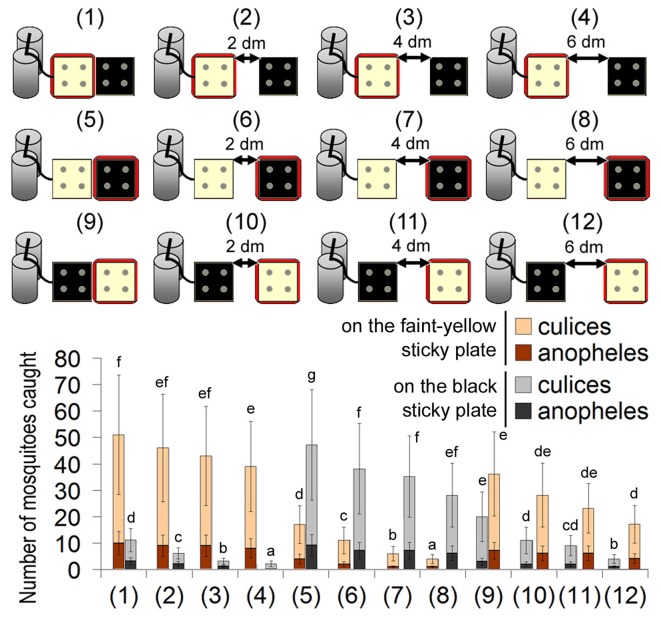
Capture rates of 12 different traps with two sticky plates of different colors. For each trap, a faint-yellow sticky plate and a black sticky plate were placed together at an interval of 0, 2, 4 or 6 dm. One of them was heated to 37.0°C (indicated by a red square frame), and the other one was not heated. Error bars show standard deviations (*n* = 5). Significant differences are indicated by different lowercase letters for each color of sticky plate.

In most cases of animal hosts, these three cues (CO_2_, heat and sometimes the black color) co-exist in the same place. What happens if one cue is separated from the others? When the CO_2_ and heat source were combined, while the black object was separated, most mosquitoes landed on the heat source. Only 10% of the mosquitoes landed on the black object nearby (Figure 4). If either the CO_2_ or the heat source was separated from the others, mosquitoes still explored the heat source, irrespective of its color (Figure 4).

## Discussion

Indole (although it is an attractant for flies; Gonzalez et al., 2015) and DEET (Ditzen et al., 2008; Lee et al., 2010; DeGennaro et al., 2013; Stanczyk et al., 2013) are mosquito contact-repellents, which inhibit the approach of female mosquitoes to hosts. Mosquitoes are repelled within 60 ms of contact with DEET-treated skin. However, a large number of mosquitoes were still caught on the sticky plates in the presence of either indole or DEET (Figure 1D), which may be because the chemicals were not sprayed on the sticky plate directly, but placed 0.5 cm below the sticky plate.

Many studies have demonstrated that mosquito females use vision to actively orient towards black objects (Bidlingmayer and Hem, 1980; Browne and Bennett, 1981; Muir et al., 1992; Gibson and Torr, 1999). It is interesting that mosquitoes can discriminate not only black and white, but also maybe different colors. A field study showed that about 44% of mosquitoes were trapped on diode-equipped sticky cards fitted with green light-emitting diodes (LEDs). Significantly more females of *Aedes* and *Culex* were captured by blue LEDs compared with red or infrared LEDs. Sticky cards with blue LEDs captured significantly more *Culex* females than those with infrared LEDs (Bentley et al., 2009). However, additional work is still needed to answer how the light wavelength composition influences mosquito behaviors.

A previous report demonstrated that mosquitoes had a significantly stronger response to moist and warm objects at distances of 6–8 cm, rather than the short 2 cm region above the floor in which mosquitoes responded to the heat plume without water (van Breugel et al., 2015). This observation suggested that the secondary effect of humidity may be an additional cue. This orientation behavior would help mosquitoes differentiate warm radiant objects (such as dark rocks heated by the sun) from animals, which increase the humidity around them when they perspire (Burgess, 1959; Eiras and Jepson, 1994). The wet heat plumes would likely disperse more slowly than the dry heat plume, however they resulted in similar capture rates. Thus, humidity may affect the detection distance, but probably not mosquito landing.

McMeniman et al. (2014) found that CO_2_ evokes mosquito’s heat-seeking behaviors, and van Breugel et al. (2015) showed that CO_2_ influenced visual target seeking. Both of these studies suggest that CO_2_ can gate responses to other sensory stimuli. Our field study also showed that, for the mosquito species tested, CO_2_ is the most important cue (prerequisite) for the landing. A previous study indicated that cyclopentanone and pyridine mimic the electroantennogram effects of CO_2_, and therefore are able to attract mosquitoes in large numbers in the absence of carbon dioxide (Tauxe et al., 2013). Here in this study, it is interesting to observe that cyclopentanone and pyridine lured mosquitoes but did not lead to landing. Mosquitoes may be attracted by CO_2_ analogs, but they can discriminate CO_2_ from other compounds and therefore do not land. There appear to be some differences in electroantennogram responses to CO_2_, cyclopentanone and pyridine stimulations (Tauxe et al., 2013).

For all odorant chemicals used in this study, no one can replace or completely inhibit the role of CO_2_ in mosquito landing, except for meperfluthrin. Use of meperfluthrin, permethrin or other pyrethroids (Xue et al., 2012) may be the most simple and feasible anti-mosquito strategy. Although the degree of cytotoxicity of pyrethroids on human cells is much lower than on insect cells (Yun et al., 2017), these insecticides induce significant increases in sister chromatid exchanges (therefore showing some genotoxic effects) and cause apparently oxidative stresses in cultured human lymphocytes in a dose-dependent manner (Azab et al., 2017). More efficient and safer pyrethroids should be developed (Haverinen and Vornanen, 2016).

## Author Contributions

SY designed the study and wrote the article. YH-Z, Z-WZ, Y-FF and G-CZ performed the research. All the authors analyzed the data, discussed the results and made comments on the manuscript.

## Conflict of Interest Statement

The authors declare that the research was conducted in the absence of any commercial or financial relationships that could be construed as a potential conflict of interest.
