# Supplementary material for: Carbon Dioxide, Odorants, Heat and Visible Cues Affect Wild Mosquito Landing in Open Spaces
Source: Front Behav Neurosci. 2018 May 7;12:86. doi: 10.3389/fnbeh.2018.00086 (PMC5949359; doi:10.3389/fnbeh.2018.00086)
Supplement: Supplementary file 1 [file Image_1.PDF]

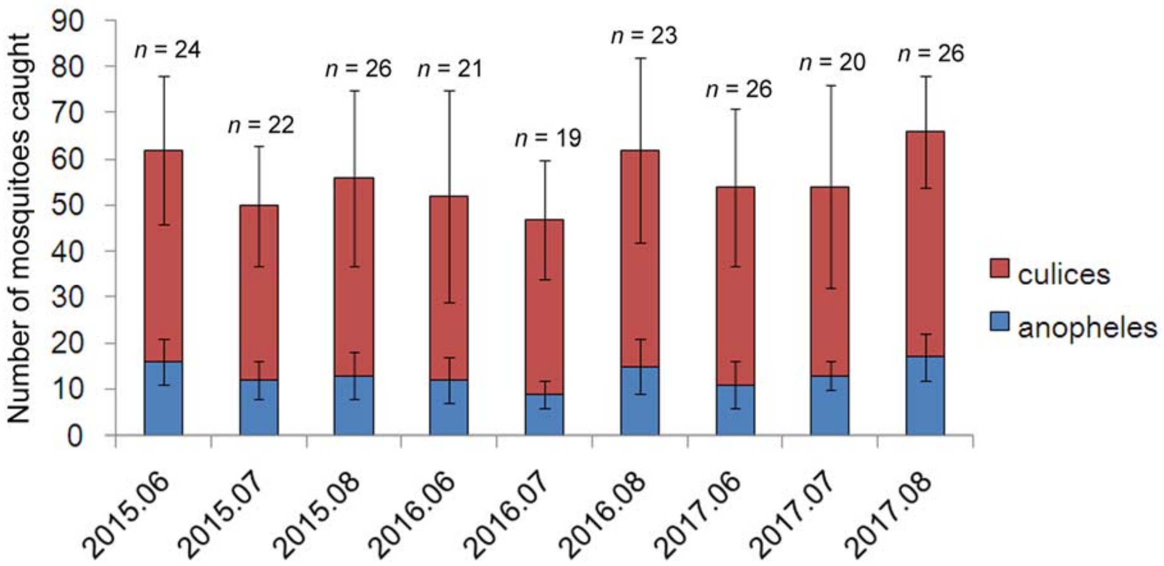

FIGURE S1 | Variations of number of mosquitoes caught by the basic trap in different months and different years. The basic trap should catch 9-17 *Anopheles* and 30-56 *Culex* per night (8:00 p.m. to 8:00 a.m.). The "*n*" value indicates the number of calm and rainless days met this criterion each month. From June 1st to September 1st in 2015, 2016 and 2017 (279 days), there were 207 such days. Error bars show standard deviations. No significant differences could be found among different months or different years.

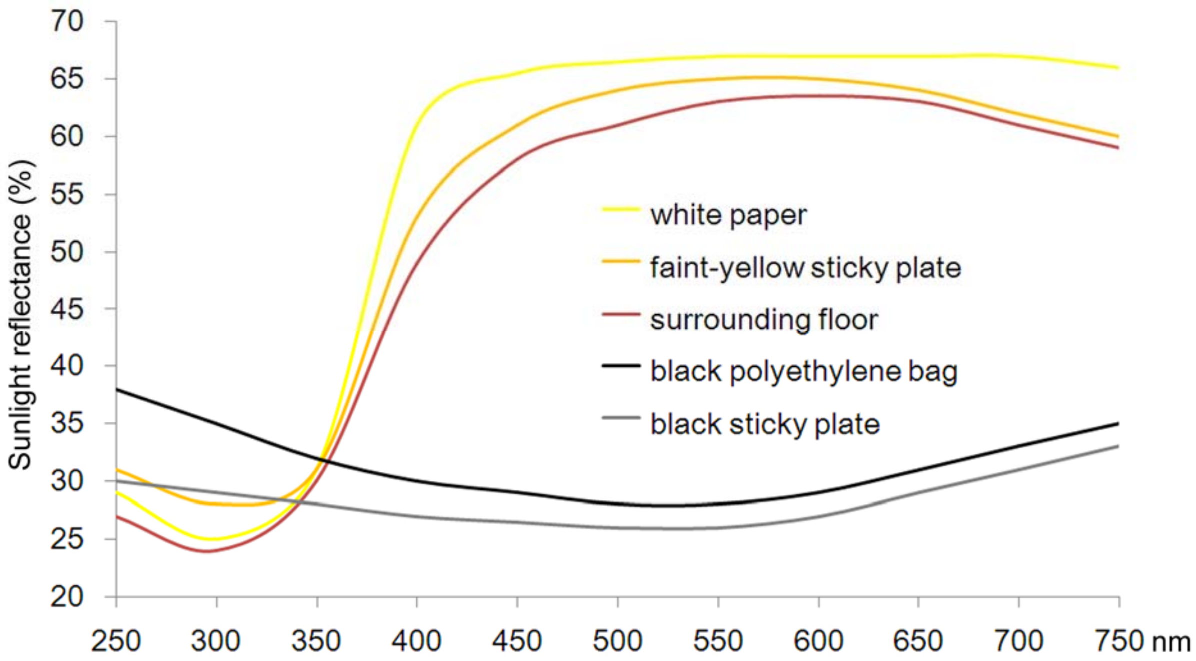

FIGURE S2 | Sunlight reflectance of different materials used in the study. The reflectance across 250-750 nm range for the faint-yellow sticky plate, the black sticky plate, the white paper, the surrounding floor and the black polyethylene bag was measured.
